# The role of prehabilitation in HNSCC patients treated with chemoradiotherapy

**DOI:** 10.1007/s00520-024-08834-3

**Published:** 2024-09-05

**Authors:** Riccardo Gili, Sacco Gianluca, Archetti Paolo, Simoni Federica, Lovino Camerino Paola, Caprioli Simone, Sarocchi Matteo, Bacigalupo Almalina, Marchi Filippo, Del Mastro Lucia, Stefania Vecchio

**Affiliations:** 1 Medical Onclogy, Department of Internal Medicine and Medical Specialties, University of Genova, Largo Rosanna Benzi 10, 16132, 16100 Genoa, Italy; 2 Medical Oncology Unit, IRCCS Ospedale Policlinico San Martino, 16132 Genoa, Italy; 3 Cardiovascular Disease Unit, IRCCS Ospedale Policlinico San Martino, Genoa, Italy; 4 Unit of Otorhinolaryngology-Head and Neck Surgery, IRCCS Ospedale Policlinico San Martino, Largo Rosanna Benzi, 10, 16132 Genoa, Italy; 5 Department of Surgical Sciences and Integrated Diagnostics (DISC), University of Genoa, Viale Benedetto XV, 6, 16132 Genoa, Italy; 6 Radiology Unit, IRCCS Ospedale Policlinico San Martino, 16132 Genoa, Italy; 7 Radiation Oncology Policlinico San Martino IRCCS, Genoa, Italy

**Keywords:** Head and neck squamous cell carcinoma, QoL, Prehabilitation

## Abstract

**Background:**

Radiotherapy (RT) is used in head and neck squamous cell carcinoma (HNSCC) with excellent effectiveness, but it is burdened by important side effects, which may negatively impact patients’ quality of life (QoL). In particular when associated with chemotherapy (CT), that has a radiosensitising effect (and its own toxicities), it is responsible for several adverse events, causing social discomfort and lower QoL, in patients who are already experiencing several tumor-related discomforts. Prehabilitation is a healthcare intervention consisting of several specialist visits prior to the start of treatment, with the aim of improving the patient’s health status, resolving symptoms that interfere with treatment and impact QoL, and finally to better avoid or overcome complications. Of all cancer patients, HNSCC patients are among those who could benefit most from prehabilitation, both because of the high number of symptoms and toxicities and their difficult management. Despite this and the emerging data, prehabilitation is not often considered for the majority of patients undergoing (C)RT. In this review, we tried to understand what are the main areas in which interventions can be made prior to the (C)RT start, the possible side effects of the treatment, the effectiveness in their prevention and management, and the impact that prehabilitation may have in adherence to therapy and on the principal survival outcomes, providing important guidance for the planning of future studies.

**Evidences and conclusions:**

Although there is no strong data evaluating multidisciplinary prehabilitation strategies, evidence shows that optimizing the patient’s health status and preventing possible complications improve the QoL, reduce the incidence and severity of adverse events, and improve treatment adherence. While cardiology prehabilitation is of paramount importance for all patients undergoing concomitant CRT in the prevention of possible side effects, the remaining interventions are useful independently of the type of treatment proposed. Geriatricians have a key role in both elderly patients and younger patients characterized by many comorbidities to comprehensively assess health status and indicate which treatment may be the best in terms of risk/benefit ratio. Collaboration between nutritionists and phoniatrics, on the other hand, ensures adequate nutritional intake for the patient, where possible orally. This is because optimizing both body weight and muscle mass and qualities has been shown to impact key survival outcomes. Finally, HNSCC patients have the second highest suicide rate, and the disease has side effects such as pain, dysfiguration, and sialorrhea that can reduce the patient’s social life and create shame and embarrassment: A psychological intake, in addition to the usefulness to the patient, can also provide current support to caregivers and family members. Therefore clinicians must define a personalized pathway for patients, considering the characteristics of the disease and the type of treatment proposed, to optimize health status and prevent possible side effects while also improving QoL and treatment adherence.

## Introduction

Head and neck squamous cell carcinoma (HNSCC) is a heterogenous group of tumors that arise from oral cavity, oropharynx, nasopharynx, hypopharynx, or larynx. It is the sixth most diagnosed cancer worldwide [1], with approximately 800,000 new cases and 400,000 deaths annually [2]. The highest incidence occurs among males aged 50–70 years, although the geographic distribution of specific subsites varies significantly [3]. The prevalence of HNSCC is strongly associated with smoking and alcohol consumption. However, in recent years, a remarkable increase of oropharyngeal cancers among younger patients was observed primarily caused by chronic human papillomavirus (HPV) infection [4]. Despite advancements in multimodal treatments and imaging techniques, the overall survival rate for HNSCC patients has not significantly improved due to the high rate of disease recurrence and metachronous tumors [5]. In case of advanced HNSCC, therapeutic strategies include surgery ± adjuvant radiotherapy (RT) (alone or combined with chemotherapy) and concomitant or sequential chemo-radiotherapy (CRT). All regimens are burdened by important side effects, which may negatively impact patients quality of life (QoL), in addition to tumor-related complications. CT has its own specific toxicity which depends on the type of drug administered [6, 7], and when it is administered in combination with RT, it has a radiosensitizing effect, increasing the typical adverse events of RT such as mucositis, sialorrhea and/or xerostomia, pain, dermatitis, dental problems, trismus, dysphagia, and concomitant weight loss, causing social discomfort and lower QoL [8]. Prehabilitation, in particular in organ-preservation regimens, is a form of healthcare intervention consisting of several specialist visits prior to the start of treatment. Prehabilitation in HNSCC patients has two different roles: to improve the patient’s state of health and to implement those measures necessary to reduce the possible CRT toxicities, considering the harm-benefit ratio that treatment may entail. For HNSCC patients, it is important, before starting RT/CRT, to perform specialist examinations to assess the health status, the presence of pre-existing and/or tumor-related problems and to implement preventive measures to reduce possible treatment-related side effects. It is also important to identify different risk classes for adverse events that can guide medical oncologist and radiation oncologist on the most appropriate treatment for each patient, for an increasingly personalized medicine. Despite this, unlike rehabilitation, prehabilitation is not often considered for the majority of patients undergoing (C)RT. The aim of this review is to understand what are the main areas in which interventions can be made prior to the (C)RT start, the possible side effects of the treatment, the effectiveness in their prevention and management, and the impact that prehabilitation may have in adherence to therapy and on the principal survival outcomes, providing important guidance for the planning of future studies.

## Psychological prehabilitation

It is well known that surgery, RT, systemic treatments, or their combinations in locally advanced HNSCC is exhausting and may cause permanent functional limitations. In particular, for concomitant and sequential CRT, swallowing, speech, and respiratory functions are usually severely affected, sometimes permanently [9]. Oral or esophageal mucositis often occurred during CRT, complicating food intake and nutritional status, already difficult in most cases by tumor-related pain and obstruction, necessitating the use of nasogastric tube (NGT) or percutaneous endoscopic gastrostomy (PEG) to save sufficient intake of nutrients, exposing the patient to an additional physical and mental stress.

Immediate side effects may persist beyond the treatment period, potentially becoming permanent or delayed consequences of the therapy, but some delayed effects might also manifest after 90 days from the end of the treatment. Delayed effects can have severe physical, emotional, and social implications [10], including difficulties such as swallowing issues, pain, xerostomia, heightened mucosal sensitivity, dysgeusia, and dental problems [11]. These effects are widespread and affect the ability to consume food and beverages. Consequently, eating and the entire dining experience could remain problematic for extended periods, stretching into months or even years. For some patients normal eating patterns might never be fully restored [12, 13]. The delayed repercussions of treatment can also contribute to physical, functional, nutritional, and psychosocial side effects, all of which have a bearing on the overall quality of life (QoL) [14, 15].

Diagnosis and treatment of HNSCC are emotionally and physically stressful periods for patients with high risk of social withdrawal [16] and depression [17], involving a higher risk of suicide [18]. Moreover, HNSCC patients use to face smoke addiction and alcoholism, typical risks factors for clinical depression and suicide. Greater depressive symptoms are also associated with shorter survival (hazard ratio, 0.868; 95% confidence interval [CI], 0.819–0.921; *P* < 0.001), higher rates of CRT interruption (odds ratio, 0.865; 95% CI, 0.774–0.966; *P* = 0.010), and poorer treatment response (odds ratio, 0.879; 95% CI, 0.803–0.963; *P* = 0.005) [8]. Retrospective analysis in Taiwan showed how HNSCC does not have a high mortality all-cause rate, compared to other tumors, such as pancreatic, esophageal, lung, or liver cancer, but HNSCC is related with a higher suicide mortality risk compared to all other tumors [89]. More specifically, HNSCC patients are second only to esophageal cancer patients in terms of suicide rates [19] also due to the important treatment side effects and the role the disease plays on patients’ quality of life (QoL). Even if it is a retrospective study, suicide mortality rate seems not related with the oncological poor prognosis than with the patient’s QoL strongly affected by treatment side effects and the complications caused by the tumor (pain, dysphagia, facial disfigurement, speech difficulties). Cancer diagnosis forces HNSCC patients to face physical, psychological, social, and emotional issues and cause dramatic consequences in their way of living and disease acceptance. Anxiety, depression and agoraphobia may compromise patient’s QoL underwent to radical and successful multimodal treatments. Psychological prehabilitation, carrying on during treatment and at least the first follow-up period, may result in an important tool in terms of prevention for HNSCC patients who are eligible for radical treatment.

The role of the psychologist is fundamental to the patient’s care pathway, and the taking charge of the patient must take place at the same time as the clinicians, educating them for a good doctor-patient relationship. This helps the understanding of what the patient will face, the possible side effects, and their management [20].

A standard psychological approach is not possible due to the variability of patients, notwithstanding that the psychologist can work on different aspects as experiential avoidance, posttraumatic growth, hope and optimism, acceptance, and commitment, interfacing also with relatives particularly in the most sensitive cases (young patients, presence of young children) [21]. Experiential avoidance (EA) is defined as attempts to avoid thoughts, feelings, memories, and physical sensations; a low EA indirectly predicts a higher QoL among cancer patients via its effect on depression symptoms [22].

Posttraumatic growth (PTG) describes positive psychological changes experienced as the result of a struggle due to a life-threatening crisis or event and comprises five components: who has experienced more PTG will better appreciate life and have better interpersonal relationships. PTG results in positive psychological changes beyond pre-trauma levels, and it is important because it inversely correlates with depression and psychological distress [23].

Hope is a positive, goal-directed motivational state and a dispositional trait that enables a tendency to adopt a positive outlook on life, and similarly, *optimism* is the stable and consistent belief that good things—rather than bad things—will happen in life. They are positively correlated with psychological well-being and QoL and inversely correlate with depression and psychological distress [24]. Acceptance and commitment therapy (ACT) is a third-generation cognitive behavioral approach that uses acceptance and mindfulness processes, as well as commitment and behavioral change processes, to generate psychological flexibility, and useful for cancer patients because it can help them handle the negative emotions resulting from cancer (such as uncertainty, anxiety, sadness, and anger), rather than avoiding these emotions [25].

## Cardiological prehabilitation

There is a significant overlap in risk factors for HNSCC and cardiovascular (CV) events; hence, many of these patients have a high CV risk. This is noteworthy as these factors are known to increase surgical risk [26]. In this setting, in accordance with the most recent guidelines on CV prevention and non-cardiac surgery management [27, 28], cardiologists take a leading role by assessing the patient’s CV risk factors and history. By identifying those who require the most attention, cardiologists aim to lower cardiovascular risk as much as possible, without interfering with the surgical and oncologic programs. Thorough CV evaluation with the aid of an electrocardiogram and echocardiogram during cardiological consultation is important as these tools can provide valuable insight for treatment selection. An ECG can easily help uncover unknown CV disease such as silent myocardial infarctions, overload, or conduction and rhythm disturbances. Moreover, even unremarkable findings are helpful as they set a base for future comparison. Routinary echocardiogram is not mandatory, but becomes useful for further investigating high risk patients, eventual pathological ECG findings, or symptoms referred during the consultation.

Finding a previously unknown heart disease is not desirable, but the setting in which it is discovered gives patients the opportunity to undertake the proper diagnostic-therapeutic path in a stable setting, avoiding unnecessary delays while maintaining optimal care. Moreover, by incorporating these diagnostic tools, clinicians can make informed decisions regarding cancer treatment options and tailor therapies to the individual’s cardiovascular needs.

Risk stratification may benefit from Supra Aortic Trunks (SAT) echo-imaging too, as presence of carotid plaque or stenosis further increases CV risk. In a risk-lowering approach, identifying SATs atheromasia highlights the need of LDL-cholesterol treatment. Furthermore, radiotherapy is involved in HNSCC treatment, and while crucial, it can generate reactive oxygen species (ROS) and, inevitably, ROS-related damage [29]. Radiation exposure may lead to new-onset peripheral artery disease and worsening of pre-existing conditions [30]; considering that HNSCC develops in the area close to carotid arteries, this further enhances the importance to conduct SATs echographic screening before radiotherapy. Lipid-lowering therapy usually is not the main focus in cancer patients but should be prompted to reduce plaque instability and prevent disease progression, ultimately helping reduce CV risk. External-beam thyroid irradiation is known to cause hypothyroidism. On the other hand, an increase in thyroid hormone levels is uncommon, but has a significant impact. Therefore, it may be useful to assess thyroid hormone levels before initiating treatment. From a cardiovascular perspective, even transiently elevated blood levels may contribute to the development of atrial fibrillation of which implications will be discussed shortly. To better understand why CV risk evaluation is crucial, fluorouracil (5-FU) constitutes a significant example. The role of fluorouracil therapy in HNSCC treatment is significant; however, its impact on the cardiovascular system cannot be disregarded, especially in HNSCC patients who tend to have a high CV risk. Because this treatment can cause vasospasm involving coronary arteries with non-critical lesions, clinicians must inform patients of the characteristics of typical cardiac chest pain and the need to contact emergency services in the event of precordial pain [8]. Although not considered among the drugs with the highest CV toxicity, cisplatin is administered with plenty of hydration to prevent renal damage. A cardiological evaluation is therefore necessary to assess the occurrence of possible heart failure. Immunotherapy has emerged as a promising treatment for HNSCC, particularly with the use of immune checkpoint inhibitors (anti-PD 1), such as pembrolizumab and nivolumab. They offer a favorable safety profile compared to traditional chemotherapy agents [31]. However, it is important to note that immune-related adverse events can occur: although relatively rare, cardiotoxicity such as myocarditis and pericarditis have been reported. While no real preventative measures are available, patients may benefit from a screening strategy through troponin dosage before and after the first 4 administrations. The rationale, while being controversial, lays behind the theory that an increase in troponin may be an indication of myocarditis development. Without a baseline value, however, troponin findings during immunotherapy administration may be misinterpreted [31].

### Lifestyle improvements and counseling

Lifestyle habits have a strong impact on prognosis, leading to poor cardiorespiratory fitness, heightened symptom burden, and consequently poorer clinical outcomes. HNSCC patients often encounter a worsening of physical capabilities during treatment. In addition, a reduced functional capacity (METS < 4 or DASI score < 34) is associated with poorer CV outcome in non-cardiac surgery [28]. This underlines the need to improve upon what effectively is a modifiable risk factor. It seems reasonable to adopt a rehabilitation-like approach by establishing a personalized schedule, generally involving aerobic exercise, and obtaining as much progress as possible while remaining tolerable. Benefits derived from this approach seem to reduce surgery-related impairments and complications, lengths of hospital stay [32], and overall chemotherapy tolerability [32]. Furthermore, cancer patients experience a pro-thrombotic state, usually in the form of venous thromboembolism. Physical activity may help in this setting as it has been linked to a decrease in the occurrence of VTE [33].

Smoking and alcohol consumption are widespread among HNSCC patients: Studies suggest that these habits involve between 60 to 90% of patients [34]. These habits must be halted considering they hinder cardiopulmonary function recovery and make the prognosis worse overall [34]. Cardiologists can take part in this process, but counseling with other specialists remains crucial for patient adherence.

### Pharmacological remodeling

As previously stated, HNSCC patients tend to have a high CV risk, but may overlook the management of risk factors. Hypertension, if approached on time, can be properly managed. Indicatively blood pressure values should remain lower than 135/85 mmHg for patients under 65 years old, while a more conservative target (140–150/90 mmHg) may be sufficient for older patients to avoid perioperative hypotension. Prompt identification of elevated blood pressure in the prehabilitation setting allows clinicians to begin therapy at least 15 days before surgery, as recommended, giving enough time to titrate therapy-based patient response. Failing to identify hypertension in time may lead to aggressive anti-hypertensive drug dosages and subsequent hypotension before or, in the worst-case scenario, during surgery. On the other hand, missing hypertension altogether may result in discovering high blood pressure directly in the operating room; in some cases, surgery may be postponed, and in cases where surgery is performed, the patients are exposed to a higher risk of cardiac overload, neurological strain, and bleeding [35]. When pharmacological therapy is needed, in absence of contraindications (e.g., CKD, ion profile alterations), ACEi/ARBs are usually preferable to other medications, especially if CV disease is present; in addition, considering they have a key role in heart failure therapy, they may be useful by mitigating some of the negative side effects of commonly used chemotherapy drugs [36]. Platin and taxanes are two of the recurrent drugs used in HNSCC treatment; among other side effects, they can lead to heart failure and fluid retention; this may justify preventively switching other anti-hypertensive drugs to ACEi/ARBs. Moreover, acting on the renin-angiotensin system seems to reduce oxidative stress, another harm mechanism potentially linked to chemotherapy-related heart failure [37]. Chemotherapy treatments can lead to the development of arrhythmias, which typically manifest as an increase in premature ventricular contractions (PVCs), atrial fibrillation (AF), and elevated heart rate. Fortunately, life-threatening arrhythmias such as ventricular tachycardia (VT) are rare occurrences [34]. To alleviate symptoms and decrease myocardial oxygen consumption, beta-blockers can be beneficial by inhibiting the adrenergic system. These medications are prescribed as part of standard chronic heart failure treatment, offering potential cardio-protective effects.

In cases where AF is present, the administration of anti-arrhythmic drugs and anticoagulants may be necessary, this adds complexity and co-morbidity to an already intricate patient condition. Due to the potential pro-arrhythmic risk of ion-imbalance in the postoperative period and during chemotherapy, rhythm control with class-IC antiarrhythmics should be carefully re-considered. In addition, nDHP treatment with verapamil should be re-evaluated due to potential drug-drug interactions with CT, mediated by CyP450 [38].

On the other hand, although direct interactions between common chemotherapic drugs for HNSCC (such as platinum, taxanes, and fluorouracil) and direct oral anticoagulants (DOACs) have not been reported, it is important to consider certain factors. The aforementioned chemotherapeutic drugs may cause myelosuppression and consequent anemia, meaning that DOAC initiation has to be carefully thought out; additionally, thrombocytopenia can be present, prompting once again for a careful evaluation and interruption if platelets levels reach under 50 k. To better understand this problem, even for patients without ongoing anticoagulant therapy, acute bleeding can still be a severe and life-threatening complication, with reported incidences ranging from 0.5% to 10%. Patients experiencing massive bleeding can be at risk of hemorrhagic shock and airway obstruction [39].

The management of dual antiplatelet therapy (DAPT) and direct oral anticoagulants (DOACs) should not be overlooked: Drug regimen modifications should be carefully considered due to potential consequences and complexities. It is essential for cardiologists and oncologists to collaborate and adhere to the latest guidelines when making decisions regarding treatment regimens. As an example, DOAC interruption should be as short as possible in patients with previous stroke or embolism (highest CHADSVASC score).

Clinicians must also remember that DOACs do not require low-molecular-weight heparin (LMWH) shift before surgery; when necessary, like in non-compressible locations, the timing of suspension can be determined using readily available charts. Interruption time is based on the bleeding risk related to the specific kind of procedure. In addition, the distinction between halting therapeutic-dose anticoagulation and the necessity of introducing VTE prophylaxis should be clarified to practitioners. Table 1 reported the CV interventions necessary for the correct risk assessment and management for HNSCC patient.

**Table 1:** CV interventions for the correct risk assessment and management for HNSCC patient

What	How	Why
Cardiovascular screening	ECG	- Aids discovery of covert CV disease - Helps in the management and finding of arrhythmias and conduction disturbances - Baseline data may be useful for future confrontation
	Echocardiogram	- Define suspected cardiopathy or to investigate high risk patients - Explore the patients’ condition if CV disease is already known
	SATs echo	- Better define CV risk - Identify patients more at risk for disease development/worsening, such as those exposed to RT - Initiate eventual therapy for plaque stabilization
	TnI HS	- High CV risk/known CV disease increases myocarditis risk during immunotherapy. Baseline dosage aids in patient management during treatment - Knowing pre/post op values helps in high risk patients’ management
	Bloodwork	- RT exposure may lead to hormonal imbalance and AF, baseline thyroid hormones level may be helpful - Basic blood count may be useful for future confrontation
Lifestyle improvements and counselling	Physical activity	- Improves baseline physical status before surgery and CT, ultimately helping recovery and tolerability - Reduces VTE
	Alcohol consumption and cigarette smoking	- Interruption improves respiratory status, reduces surgical risk and favors recovery
	Chemotherapy CV side effects	- Informed patients benefit from knowing significant side effect and when to seek for help
Pharmacological interventions	Blood pressure medication	- Controlled blood pressure reduces CV risk - Timely hypertension addressing simplifies management and reduces over/under treatment - ACEi/ARBs may help reduce CT cardiotoxicity
	Rhythm and heart rate control	- Beta blocker reduce arrhythmias and PVCs while hindering CT cardiotoxicity - Class IC antiarrhythmics can have serious side effects due to ion alterations and surgery induced stress - nDHP calcium blockers have common drug-drug interaction
	Drug interactions and possible contraindications	- CT may have serious drug-drug interactions - Surgery-related stress and ion alterations may cause drug-related complications
	DAPT and DOACs management	- Suspension timing must be thought out to reduce surgical bleeding - Abrupt and prolonged DAPT/DOAC interruption is unadvised - VTE prophylaxis cannot be overlooked - High-bleeding risk patients need close follow-up and possible discontinuance

In conclusion, cardiovascular prehabilitation in cancer treatment is decisive and gives patients the opportunity to tackle issues proactively. Recent guidelines on prevention and perioperative management stress the importance of bringing the patient to surgery with the optimal CV risk. This is especially true for HNSCC patients, due to the frequent combination of high pre-existent CV risk and complex radical surgery and adjuvant treatment. Hence, a dedicated cardiovascular evaluation in the prehabilitation scheme should be implemented.

## Nutritional prehabilitation

In HNSCC patients the prevalence of malnutrition at diagnosis can range from 42 to 77%, and it usually worsens during treatment [40]. (C)RT is associated with high rates of toxicities and complications: Up to 90% of HNSCC patients treated with RT alone or CRT develops acute nutrition impairment due to the symptoms caused by patient’s lifestyle, tumor location, and treatment toxicities, such as pain, mucositis, dysgeusia, xerostomia, and dysphagia [41].

Weight loss correlates with poor overall condition, reduced tolerance and adherence to treatment, and consequently to the risk of complications and worse prognosis [42]. In addition to body weight measurement and its variation, the evaluation of the body composition is paramount; in particular, sarcopenia (reduced skeletal muscle) is a crucial factor in HNSCC patients that predicts clinical outcomes and treatment toxicities [43, 44].

Sarcopenia (diagnosed by measurements of muscle strength and muscle quantity and/or quality) is often a consequence of malnutrition and is independently associated with mortality in patients with advanced solid tumors [45], with an increasing risk of death across the continuous distribution of Skeletal Muscle Index (SMI), regardless of sex, age, HPV status, tumor site, performance status, and in particular in patients without clear symptoms of malnutrition or depletion [46]. Patients affected are characterized by lower muscle density, indicating an alteration in muscle quality with an increase of intramuscular fat infiltration and deposit due to the pathologic redistribution of adipose tissue, and it is associated with chronic inflammation and impaired glucose tolerance [44]. Preserving muscle quantity and quality with proper nutritional support, both before and during (C)RT, may impact positively on HNSCC patients survival outcomes [47]. In addition, the cancer-related inflammatory status, by circulatin cytokines release, could be a key driver of malnutrition, and it is accompanied by metabolic effects, such as insulin resistance and reduction of appetite [48]. Merker et al. evaluated the association between the inflammatory status (misured with C-reactive protein) and the nutritional support, confirming the role of systemic inflammation on the patients’ prognosis and the cytokine-induced downregulation of food intake [48].

Because in many trials not all patients had the same response to supportive nutrition, it is assumed that inflammatory status, causing metabolic changes, could play a key role in its effectiveness, making personalized supplementation regimens necessary [48].

In patients undergoing CRT, the nutritional intervention is required at three different times: at diagnosis, during therapy, and in the first follow-up. Early nutritional assessment is useful to optimize patient’s nutritional status and to minimize the impact of acute toxicities and treatment interruptions, to reduce unplanned hospital admissions and the incidence of infections, and ultimately to improve survival [49], while close nutritional monitoring during treatment is necessary to tailor the nutritional support to the emerging toxicities.

Before starting treatment, nutritional assessment is essential to understand whether the patient is malnourished, and during CRT, it is also recommended every 2–3 weeks (adjusted according to the patient's needs), and it should continue after the end of therapy, until nutritional status is restored [50]. Well-nourished patients only need diet-counselling, while in malnourished patients, nutritional intake is assessed. When patients have a nutritional intake greater than 75% of nutritional requirements, they are referred to dietetic counseling and nutritional supplements; otherwise, enteral nutrition via NGT or PEG is indicated [51]. In patients in whom PEG and NGT placement is not possible or rejected by the patient, enteral nutrition (EN) is a further option preferred for individuals with an intact gastrointestinal tract to promote maintenance of gut integrity [52].

Oral nutritional supplements are useful to supplement patient’s diet with nutrient-dense liquids, particularly for patients suffering from inappetence or for whom follow a liquid diet due to treatment toxicities [52]. There are concentrated calories and protein shakes that can be used as meal replacements, usually in two different formulations (1.5 kcal/ml or 2.25 kcal/ml).

The need for the use of these supplements varies depending on weight, age, nutritional habits, and reduced calorie intake [51]. Increasing protein intake is a predictor of oral mucositis in HNSCC [53]. With regard to EN, either NGT or PEG are excellent methods for allowing proper caloric intake. Generally, the administration of EN is largely determined by the duration of need for nutrition support and the patient's will. Generally, nasogastric tubes are used for short-term therapy (< 4 weeks). For long-term therapy (> 4–6 weeks), PEG tubes are preferred [54]. Despite their effectiveness, both NGT and PEG have critical issues. NGT can be placed for less time and is disfiguring, and PEG is a more invasive procedure and is more expensive, less disfiguring but with an increased risk of infection [53].

Enteral nutrition can be administered in three different ways (continuous, intermittent drip, or bolus) but most importantly in several formulas: standard or polymeric, elemental (containing hydrolyzed proteins reserved for patients with malabsorption or pancreatic dysfunction), or specialized [51]. Standard or polymeric formulas are suitable for HNSCC patients with intact gastrointestinal tracts: Higher caloric density formula (1.5–2.0 kcal/ml) is ideal for patients unable to tolerate large volumes, but lower caloric density formula (1.0 kcal/ml) may be easier to digest [54]. Specialized formulas designed for a particular condition such as renal disease, hepatic disease, diabetes, or pulmonary disease require documentation of medical necessity for insurance coverage and are typically more expensive [54]. Alongside the standard artificial nutrition products, immunonutrition has been deployed and is increasingly used. Immunonutrition has the aim of stimulating the host immune response, improving control of the inflammatory response, and increasing nitrogen balance and protein synthesis through immunonutrients such as glutamine; arginine; polyunsaturated fatty acids (omega-3); nucleotides; taurine; vitamins A, E, and C; beta-carotene; and trace elements such as zinc and selenium, having a protective role on potential side effects related to chemo-radiotherapy and also in development of major surgical complications [55–57]. There is still no unanimity on treatment dosage and duration in patients undergoing chemo-radiotherapy [56]. A multicenter clinical trial to address some of the most important issues is currently ongoing in Italy (registered at clinicaltrials.gov as NCT04611113).

## Geriatric prehabilitation

**Fig. 1:**
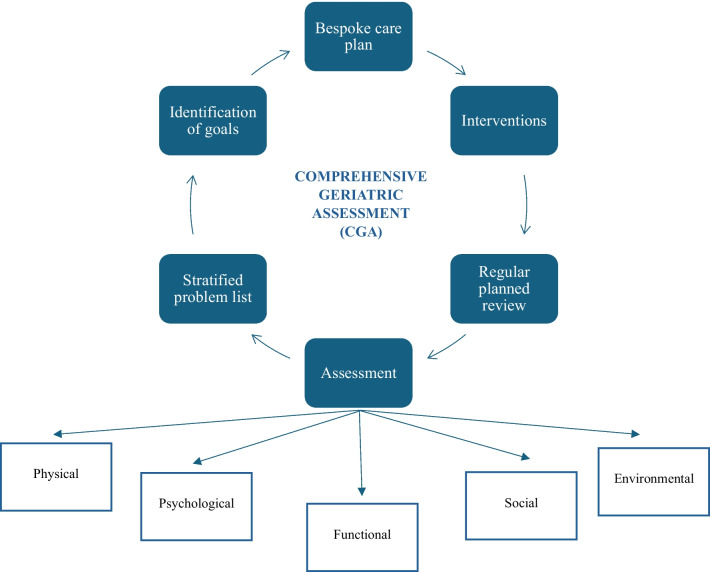
CGA diagnostic and treatment process

**Fig. 2:**
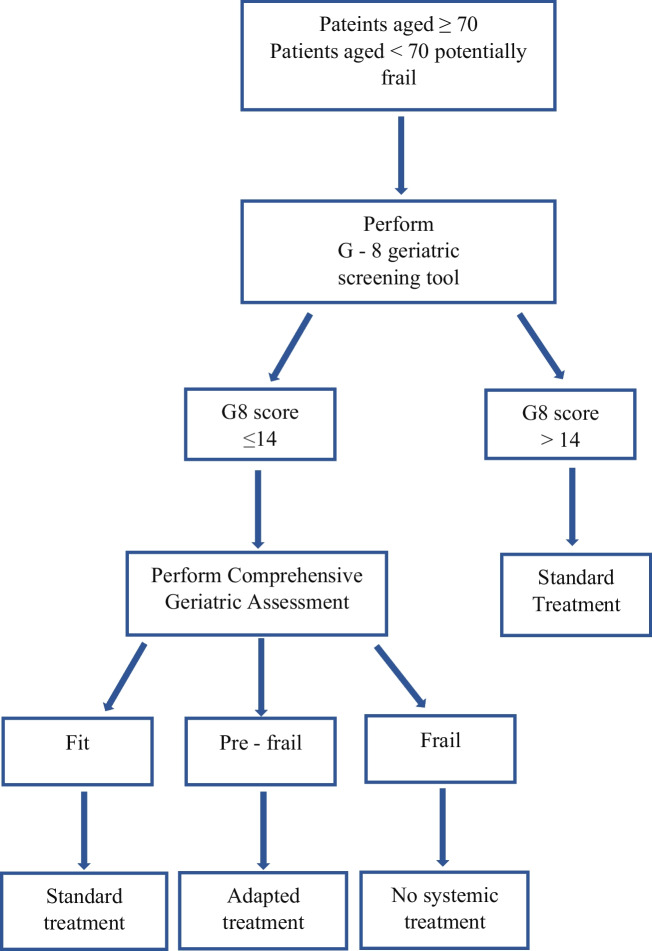
Flow chart of the geriatrician’s activities to assess the patient's state of health and to define the correct treatment

The geriatric assessment is a multidimensional and inter-disciplinary evaluation to assess the health status of patients, helping the clinician to choose the best treatment regardless of the patient’s age, playing a key role in frail patients (frailty is defined as a state of vulnerability to poor resolution of homeostasis following stress). Frail patients, and in particular for HNSCC patients, have higher rates of complications, toxicities, readmissions, mortality, and longer length of stay and are more frequently discharged to post-acute care facilities, if compared with fit patients [58]. Previously, geriatric assessment was reserved for elderly patients, normally considered to be aged > 70 [59], but Porceddu and Haddad and the European Medicines Agency consider 65 years of age the cut-off for elderly patients [60, 61]. Considering the characteristics of HNSCC patients, the usual smoking and alcohol abuse, multiple comorbidities, and the age limit of 65 years may be more appropriate for this specific patient population [62, 63]: despite comparable levels of comorbidity patients with HNSCC appear to be frailer than patients with other solid malignancies [64]. Recently, the concept of elderly/frail patients worthy of geriatric assessment has changed, no longer being related to chronological age but more to biological age, comorbidities, and health status: Frailty can also be present in younger patients aged [63]. In HNSCC patients, frailty is often accompanied by multiple impairments with serious adverse events, particularly for those cancer-related, such as dysphagia, loss of weight, pain, and disfigurement [64]. Frail patients can still undergo surgery, radiotherapy, and receive various systemic treatments with curative and palliative intent, but need tailored schedules [65]. Regarding concomitant or sequential chemoradiotherapy treatment and considering the synergistic effect the toxicities may have, prior to the final choice of systemic treatment, it is important to perform a geriatric assessment that can establish patient’s risk factors and health status, the impact of possible drug-related toxicities, and the adherence to treatment [65], with the aim of giving the best treatment strategy while preserving as much as possible QoL [66]. To establish the frailty profile of HNSCC patients is the Comprehensive Geriatric Assessment (CGA), a multidimensional battery test that analyzes comorbidity, cognition, functional status, geriatric syndromes, mood, nutritional status, polypharmacy, and social support (Fig. 1) [67]. The CGA allows the identification of 3 categories of patients: (1) fit patients who have no contraindication to receive the proposed treatment, (2) pre-frail patients for whom treatment should be modulated, and (3) frail patients who are not amenable to any active treatment: the main international societies and guidelines recommended CGA before making a decision on cancer treatment [68]. In patients aged > 65, it is also possible to define who would benefit from CGA, using the G-8 geriatric screening tool, that consists of seven items from the Mini Nutritional Assessment (MNA) questionnaire and age [69]. Patients with a G-8 geriatric screening tool score ≤ 14 benefit from CGA. However, it has recently been demonstrated that also patients aged < 65 could benefit from G-8 and subsequently from CGA, making it an assessment tool for all patients, and in particular for those with greater frailty regardless of age [64]. Figure 2 shows a flow chart of the geriatrician’s activities to assess the patient’s state of health and to define the correct treatment. Geriatricians therefore play a central role in assessing patients for both curative and palliative treatment. In particular, in addition to well-known chemotherapies such as cisplatin and carboplatin, taxanes, and fluoropyrimidines, the introduction of biologic drugs such as anti-EGFR and the immune checkpoint inhibitors (pembrolizumab and nivolumab) has changed the possible therapeutic options and the resulting drug-related toxicities. With regard to concomitant radio-chemotherapy treatments, there are 3 different drugs available: cisplatin, carboplatin, or cetuximab. Cisplatin compared with carboplatin and cetuximab has been shown to be the most effective in terms of progression free survival and overall survival, but it is burdened by major toxicities (renal toxicity, nausea, vomiting, bone marrow toxicity) that make it difficult to use in non-fit patients: It is well known that a higher incidence of acute mortality and higher rates of hospitalizations during platinum-based CRT occurs in elderly and frail patients [70]. Recent introduction of weekly (vs tri-weekly) schedules, burdened by lower toxicities, may make the use of weekly cisplatin in selected pre-frail patients worth considering [71]. For cisplatin-ineligible HNSCC, carboplatin has shown an important activity and can be a reasonable option to treat patients [72]. Although toxicities are lower than with cisplatin, it is important to remember that carboplatin can cause severe pancytopenia, so its use should be for selected patients with careful blood count monitoring. Recently, weekly carboplatin was also proposed with favorable feasibility, efficacy, and acceptable toxicity [73]. For anti-EGFR drugs, the data are still unclear. In many studies, some of which have no implication in clinical practice, the efficacy data in subgroup analyses are conflicting, while there seem to be no differences in grade 3 or higher adverse effects between the different patient groups, making these drugs well-tolerated and administered even to older and/or frail patients [74]. In the recurrent or metastatic (R/M) setting, the immune checkpoint inhibitors showed less toxicities overall, with no differences in patients of different age and frailty, and the same efficacy [74]. Thus, the efficacy of immunotherapy seems to be linked more to disease-related and host-related factors rather than age [74]. In conclusion, the geriatrician plays a key role in the management of HNSCC patients, both in detecting the most frail ones and in establishing, in agreement with the clinician, the correct management, and therapeutic strategy, also in view of the increasing life expectancy and the high rate of elderly and frail patients with HNSCC. To do this, the geriatrician must be an integral part of the multidisciplinary team and not only have a consultative role.

## Phoniatric and speech prehabilitation

It is well known that for patients who have undergone CRT, long-term irradiation of the swallowing organ at risk (SWOARs) results in glossal muscle atrophy, weakened tongue sensations, inability of the soft palate to lift, pharyngeal constrictor weakness, cricopharyngeal spasm, and other complications [75]. Treatment-induced toxicities also include mucositis, xerostomia, odynophagia, trismus, and hypogeusia/dysgeusia, and all CRT-related adverse effects can have a serious impact on the patients correct caloric intake, by reducing appetite and causing dysphagia. The acute inflammation of the mucous membranes of the pharyngeal tract represents the main dose-limiting toxicity of CRT, contributing significantly to the onset of acute dysphagia and, in some cases, even to late dysphagia [76]. The damage that leads to dysphagia mainly occurs due to three mechanisms: mucositis, xerostomia, and nerve damage. Mucositis often leads to intense pain during swallowing and in severe cases is accompanied by the abundant emission of thick filamentous secretions that can cause regurgitation and can predispose to food bolus aspiration [77]. The onset of grade 3 mucositis is associated with ulcerations in the damaged mucosal tract, resulting in a delayed healing process characterized by incomplete re-epithelialization and formation of scarred areas and fibrosis (healing by second intention), which is considered a consequential late effect. Xerostomia is related to the irradiation of the major salivary glands and typically occurs few months after the end of radiotherapy (atrophy of the serous acini appears at 16 weeks from the end of treatment), even for low doses [78]. Salivary glands are highly radiosensitive, and the destruction of salivary glands begins in the first few days of radiotherapy. In the first week, a 50% to 60% decrease in salivary flow occurs, and after 7 weeks of conventional radiotherapy, salivary flow diminishes to approximately 20%. The severity of xerostomia depends largely on the dose of radiation administered and the irradiated gland volumes. In HNSCCs, the delivered RT dose with the best benefit-to-adverse events ratio is 70 Gy, but if salivary glands receive a dose greater than 26 Gy, the salivary function can be compromised permanently [79]. Xerostomia contributes significantly to late dysphagia: Saliva plays a crucial role in achieving adequate cohesion between bolus particles. The parotid serous production is more profoundly affected by radiation treatment, resulting in increased viscosity and a reduction in the production of surface protective substances such as IgA and growth factors (EGF), which normally play a protective role in maintaining the integrity of the pharyngeal mucosa. This also leads to an increased risk of dental infections. Amifostine, a cytoprotective drug for the salivary glands with multiple side effects, can be used but only in severe cases [80]. Nervous (both sensory and motor) and vascular damage induced by radiotherapy on laryngeal tissues can result in worsening of laryngeal sensitivity with a reduced cough reflex and a greater predisposition to the aspiration of the food bolus. Dysphagia in patients with locally advanced HNSCC can be associated with the underlying disease (cancer-related dysphagia) and/or the consequences of the treatment received (treatment-related dysphagia). Among these, radiation-associated dysphagia (RAD) is of great importance. The post-RT dysphagia can be divided into acute dysphagia, which occurs during the course of radiation treatment and can last up to a maximum of 4–6 months (reversible effect), and late dysphagia, which occurs 6 months later and persists for the rest of patient’s life (irreversible effect). In addition to the well-known post-CRT speech therapy rehabilitation treatment that consists of direct postural and dietary stimulation and compensation, some authors have hypothesized that performing swallowing exercises also before the treatment could improve the functional outcome of swallowing. One of the first protocols has been proposed by the University of Alabama Birmingham (UAB), involving a sequence of various maneuvers (Masako, tongue range of motion exercises, forced swallowing, Mendelsohn maneuver, and Shaker exercises) [81]. Other exercises often performed strengthen the inspiratory and expiratory muscles, allowing an increase in hyoid range of motion. In recent years, several protocols have been developed to assess the utility of prophylactic swallowing exercises (PSE), and positive effects have been observed on muscle condition, swallowing function, and QoL. In particular, three techniques have shown an improvement in the oral, pharyngeal, and esophageal phase of swallowing after CRT [82]. The Mendelsohn maneuver is an exercise designed to treat both reduced laryngeal excursion and limited cricopharyngeal opening. This maneuver is performed by having the patient hold the larynx up, either using the muscles of the neck or with the hand, during the swallow for an extended period of time. The premise behind this technique is that if the extent and duration of laryngeal elevation could be increased, there would be a reciprocal increase in the extent and duration of the cricopharyngeal opening. In order to effectively use the Mendelsohn swallow maneuver, the patient must meet several requirements. The patient must have a sufficient amount of language ability to follow directions (therefore, a patient with severe language impairment would not be a good candidate), intact cognitive abilities, and finally good physical conditions [83]. In HNSCC who underwent CRT, when compared to the Mendelsohn maneuver, the prophylactic Shaker exercises improves patients’ swallowing function, and the thyrohyoid muscle shortening is more preserved and better close the larynx to prevent aspiration, resulting in shorter hospitalization and a significant low aspiration score [83]. The Shaker maneuver is a technique in which the patient lies flat on their back and holds their head up, holding the pose for one minute, resting for one minute, and repeating this three times. The patient can also repetitively lift their head 30 times [84]. Lastly, the Masako technique is an exercise performed by protruding the tongue between the front teeth, holding it in place by gently biting down on the anterior portion of the tongue and maintaining this posture while swallowing saliva. This technique improves the tongue-base retraction, but should not be performed with actual food or liquid because it alters the position and function of the muscles involved in the pharyngeal swallow and has been seen to result in aspiration [84]. In HNSCC patients, adherence to PSE is generally low (13%) or moderate (71%). In particular, in patients who undergone CRT, adherence significantly decreases during the 4 weeks of training, especially in patients without a close relationship with the therapist and when the use of paper-based materials or apps is preferred: Implementing the hospital and home care organization to give an extra assistance to patients is necessary to improve the therapy adherence [85]. Subclinical voice disorders are common after chemo-radiotherapy. Although patients consider vocal impairment to be very minor and not to interfere with their daily life, it may contribute to a reduced quality of life. In conclusion, all the literature works indicate that PSE can potentially reduce aspirations, improve swallowing, and restore oral intake. However, due to a lack of standardization in both the exercises used and the test procedures, as well as a lack of adherence, there are still no protocols that can provide standardized guidelines.

## Dentist prehabilitation

In HNSCC, treatment strategies can have a significant and long-lasting negative impact on oral and dental health, function, and appearance, having a detrimental effect on the patient’s QoL and psychological wellbeing. Therefore, before starting any treatment and in particular CRT, it is necessary for the patient to undergo a dental examination with the restorative dentistry, a dental oncologist specialist in the oral effects of non-surgical treatment modalities [86]. Restorative dentistry (RD) consultants provide oral and dental prehabilitation considering all relevant periodontal, endodontic, and prosthodontic factors and taking into account planned treatments and any consequences from this to provide an optimum outcome for patients [86]. RD also plays a key role in patient rehabilitation, but is especially important in the pre-treatment phase, where RD has to prepare patients optimally for cancer treatment without delay and to achieve the best to preserve patient QoL. RD prehabilitation assesses the health status of teeth and intervenes on any problems that are present (such as dental and periodontal diagnosis and extraction of teeth with poor prognosis) and may worsen due to CRT and its side effects. Evidence-based risk assessment of developing post-treatment and long-term oral and dental complications (altered anatomy, trismus, hyposalivation, radiotherapy-associated caries and osteoradionecrosis) is carried out. The same also applies to edentulous patients: They require radiographic assessment because they may have retained roots, buried teeth, or local bony pathology [86]. Regarding oral cavity, osteoradionecrosis (ORN) is the most feared side effect of CRT. ORN is a severe iatrogenic disease of devitalized bone caused by radiation therapy of oral and oropharyngeal cancers, with inadequate healing or remodeling response of at least 3 to 6 months. There is not universally accepted pathophysiologic description, ORN results from a sequence of injury events beginning with radiation exposure and exacerbated by manipulation of the affected tissues. The radiated mandible and surrounding tissues develop hyperemia, inflammation, and obliterative endarteritis. The small vessels are thrombosed, causing hypovascular-hypocellular-hypoxic tissue, which undergoes tissue breakdown [87]. ORN etiology is multifactorial. Radiotherapy indiscriminately damages cells, in particular non-tumor cells with rapid turnover and the normal osteoclast/osteoblast activity of bone healing is affected [88]. In order to prevent possible treatment side effects, all patients for whom CRT (or RT alone) is proposed after multidisciplinary discussion, particularly for oral cavity and oropharynx SCC; a dental examination with an RD is necessary. RD should be a member of the multidisciplinary team to better manage the prehabilitation of HNSCC patients who are to undergo CRT.

## Discussion, recommendations, and conclusion

HNSCC patients undergoing both surgery and (C)RT experience significant toxicities. RT either alone or in combination with CT has significant toxicities that vary depending on the site, but usually include mucositis, sialorrhea and/or xerostomia, pain, dermatitis, dental problems, trismus, dysphagia, and concomitant weight loss [6, 7]. CT on the other hand has a synergistic radiosensitizing role, which increases the effectiveness of RT but also amplifies its adverse effects. CT can also cause specific side effects, such as fatigue, myelotoxicity, nephrotoxicity, neurotoxicity, and ototoxicity in platinum-based regimens, while in 5-FU-containing regimens, cardiotoxicity is the most common adverse event [6, 7]. In addition, the tumor itself (directly or indirectly) can cause serious impairments, worsening the performance status, reducing therapeutic options and adherence to treatments, resulting in the worsening of the main survival outcomes and especially in the slowing down or inability to recover the injured functions. To decrease the incidence and severity of these events, it is necessary to select patients to receive the most appropriate treatment and to resolve pathological conditions (pre-existing or tumor-related) that may interfere with the proposed treatment. Prehabilitation is therefore the implementation of these procedures. In this descriptive review, the authors assessed what interventions may be useful prior to organ-preserving treatment in order to treat any pathological conditions that may affect the normal course of treatment and to select patients to receive the best possible treatment according to their clinical condition. While cardiology prehabilitation is of paramount importance for all patients undergoing concomitant CRT in the prevention of possible side effects (including non-drug-related toxicities as in the case of CT platinum-based) and in the detection of previously unknown morbid states, the remaining interventions are useful independently of the type of treatment proposed. It is well known how HNSCC patients are at high risk of suicide (second after pancreatic cancer patients) [89][19], due to both the symptoms caused by the tumor and the emotional impact of the diagnosis. In particular, the resulting facial disfigurement due both to the tumor and the treatments could create discomfort and embarrassment for patients, limiting their sociality, making patients depressed and isolated. The side effects of (C)RT, disease-induced dysphagia and pain, and inflammatory-related cachexia are the main contributors to weight loss and body and muscle composition and quality [42, 44], and it is already known how this impacts the main survival outcomes and treatment adherence. This is even more evident in HNSCC patients than in other malignancies, as these patients are often alone without caregivers. Individualized supplementation of oral, enteral, or parenteral nutrition (depending on tumor site, tumor-related issues, and patient compliance) [46] can improve the patient’s performance status, increase body weight, and consequently increase the efficacy of the proposed treatments and reduce the occurrence of adverse events that can lead to therapy discontinuation or serious complications, including patient death. In patients in whom swallowing is preserved, phoniatric and speech prehabilitation has a primary role both to ensure sufficient oral caloric intake and to avoid complications such as ab-ingestis pneumonia, as well as preventing CRT-induced dysphagia. Dental prehabilitation is of particular importance in patients with malignancy of the oral cavity, oropharynx, or nasopharynx, which among all possible sub-sites are those receiving more Gy during RT or CRT treatment. Indeed, RT can worsen the oral health status, and the presence of affected tissue during RT/CRT increases the risk of ORN, which is the worst of all RT/CRT-related adverse events. Even in patients with a negative history of dental disease, performing an examination and orthopantomography is recommended to exclude underlying pathologies that would pose a potential risk of developing ORN, also for edentulous patients. Finally, many HNSCC patients are elderly or have multiple comorbidities. For these patients, a multidimensional and interdisciplinary assessment is needed to evaluate their health status to choose the best treatment (regardless of the patient’s age) taking into account the risk/benefit ratio of each possible therapy, so as to propose effective individualized treatments with reduced toxicities.

## Data Availability

No datasets were generated or analysed during the current study.
